# Clinical challenges of chronic wounds: searching for an optimal animal model to recapitulate their complexity

**DOI:** 10.1242/dmm.016782

**Published:** 2014-11

**Authors:** Robert Nunan, Keith G. Harding, Paul Martin

**Affiliations:** 1 Schools of Biochemistry and Physiology and Pharmacology, University of Bristol, Bristol, BS8 1TD, UK.; 2 School of Medicine, University of Cardiff, Cardiff, CF14 4XN, UK.

**Keywords:** Animal models, Chronic wounds, Diabetic foot ulcer, Ischemia, Pressure ulcer, Venous leg ulcer

## Abstract

The efficient healing of a skin wound is something that most of us take for granted but is essential for surviving day-to-day knocks and cuts, and is absolutely relied on clinically whenever a patient receives surgical intervention. However, the management of a chronic wound – defined as a barrier defect that has not healed in 3 months – has become a major therapeutic challenge throughout the Western world, and it is a problem that will only escalate with the increasing incidence of conditions that impede wound healing, such as diabetes, obesity and vascular disorders. Despite being clinically and molecularly heterogeneous, all chronic wounds are generally assigned to one of three major clinical categories: leg ulcers, diabetic foot ulcers or pressure ulcers. Although we have gleaned much knowledge about the fundamental cellular and molecular mechanisms that underpin healthy, acute wound healing from various animal models, we have learned much less about chronic wound repair pathology from these models. This might largely be because the animal models being used in this field of research have failed to recapitulate the clinical features of chronic wounds. In this Clinical Puzzle article, we discuss the clinical complexity of chronic wounds and describe the best currently available models for investigating chronic wound pathology. We also assess how such models could be optimised to become more useful tools for uncovering pathological mechanisms and potential therapeutic treatments.

## Chronic wounds: an introduction to a global clinical problem

**Fig. 1.**
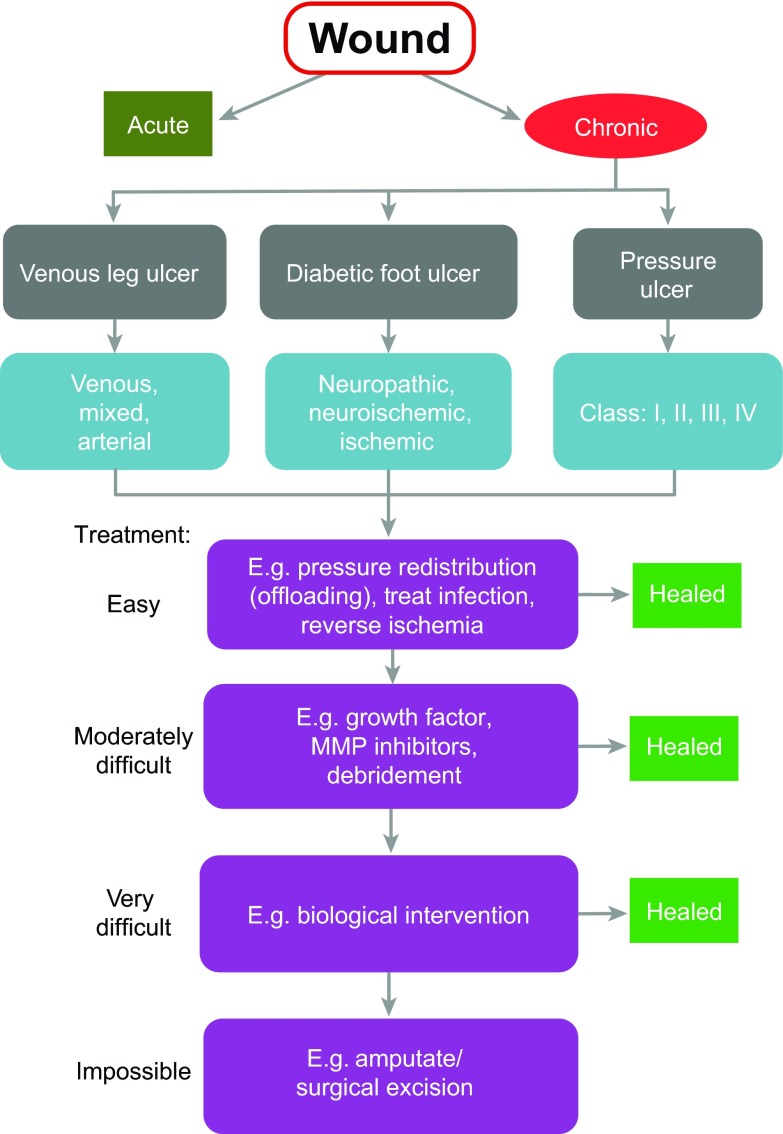
**Flow diagram of chronic wound management.** Skin lesions identified as chronic wounds are first classified into three broad categories (grey boxes): venous leg ulcers (VLUs), diabetic foot ulcers (DFUs) or pressure ulcers (PUs). Chronic wounds are then refined into sub-categories based on their suspected aetiology (blue boxes). All wounds are initially assumed to be ‘easy’ to treat and receive the appropriate care such as ‘offloading’, antibiotics or surgery. Subsequently, if healing fails, wounds receive progressively more aggressive treatment, including debridement and biological intervention. If all other treatment fails, the final resort is to amputate the affected appendage or limb.

Chronic, non-healing wounds (Box 1) can be debilitating for the affected individual and place a massive financial burden on healthcare systems. During the financial year 2005–2006, the total annual cost to Hull and East Yorkshire National Health Service (NHS) of treating chronic wounds was conservatively estimated at £15m–£18m (£2.5m–£3.1m per 100,000 population), which equates to about 2–3% of the local healthcare budget (Drew et al., 2007); these figures are likely to have escalated since this time. A recent study found a 4.2% prevalence rate of chronic wounds in a Dutch nursing-home population (Rondas et al., 2013). A typical hospital wound-healing clinic will see a large number, and a very variable range, of these lesions, but almost all chronic wounds will have a clear underlying cause and can generally be assigned to one of three clinical categories: leg ulcers, which are frequently a consequence of venous or arterial deficiencies [venous or arterial leg ulcer (VLU or ALU, respectively)]; diabetic foot ulcers (DFUs); or pressure ulcers (PUs), otherwise known as bed sores (Box 1; Fig. 1).

**Box 1. Clinical terms**

Arterial disease: lack of blood supply through the arteries; common in diabetic individuals.
Arterial leg ulcer (ALU): a leg ulcer caused by reduced arterial blood supply to the lower limb.
Biofilm: a group of bacteria that adhere to each other and produce a protective ‘slime’.
Bipedicle skin flaps: an area of tissue generated between two parallel incisions that retains a restricted supply of blood through a pedicle at each end.
Chronic wound: a full-thickness skin defect that fails to heal within 3 months.
Debridement: the act of removal of slough from chronic wounds with the aim of stimulating the healing process. This can be achieved via a variety of methods, including the use of a scalpel or larvae.
Diabetes: a group of metabolic diseases that results in high blood-sugar levels, leading to complications that include blood vessel and nerve damage.
Diabetic foot ulcer (DFU): this foot ulcer occurs in up to 15% of all individuals with diabetes and is caused by neural and vascular complications.
Fibrin cuffs: build-up of fibrin around leg veins of patients with venous insufficiency. They can reduce diffusion of oxygen to surrounding tissue.
Granulation tissue: formed during the repair process; comprised of mixed cell and tissue types, including new blood vessels, fibroblasts, myofibroblasts, matrix components and immune cells.
Hyperoxia: higher than normal partial pressure of oxygen.
Hypertension: high blood pressure.
Hypoxia: low tissue oxygen tension.
Ischemia: when blood flow to a tissue is restricted, leading to low oxygen and glucose levels.
Offloading: a treatment for diabetic foot ulcer; involves sustained pressure relief from sites of injury.
Osteomyelitis: infection of the bone.
Pressure ulcers (PUs): these ulcers [also known as decubitus ulcers or bed sores] often occur over bony prominence owing to sustained pressure and/or shear/friction forces that obstruct blood flow to the tissue.
Reperfusion injury: tissue injury caused by the return of blood supply after a period of ischemia.
Transverse rectus abdominal myocutaneous (TRAM) flap: a musculocutaneous flap of skin remaining attached to the body through a single pedicle and used to generate necrotic skin by restricting blood flow.
Vasculitis: abnormal and uncontrolled inflammation of blood vessels.
Venous leg ulcer (VLU): a leg ulcer caused by sustained levels of high blood pressure in the lower leg due to inadequate venous return.
Wound grading system (EPUAP): 1 = non-blanchable erythema of intact skin; 2 = partial-thickness skin loss, involving epidermis, dermis, or both; 3 = full-thickness skin loss involving damage to or necrosis of subcutaneous tissue that can extend down to, but not through, underlying fascia; 4 = extensive destruction, tissue necrosis or damage to muscle, bone or supporting structures, with or without full-thickness skin loss.

### Leg ulcers

Leg ulcers are a common chronic wound, with an incidence of 0.3% among the adult male population, rising to 8.3% of males over 85 (Moffatt et al., 2004). Their underlying cause can vary, but >70% are associated with venous disease, which features damage to the superficial and/or deep venous systems of the leg and consequently causes venous hypertension and reduced blood flow. This can result in the failure to heal even minor wounds to such limbs (Grey et al., 2006a). Other underlying causes of leg ulcers include arterial disease, vasculitis and skin malignancies [there is a well-established association between squamous cell carcinomas and chronic skin wounds (Enoch et al., 2004)]. Currently, the main treatment for VLUs is compression bandaging, which controls swelling and limits venous hypertension in the limb. This treatment can produce 70% ‘cure’ rates when employed in dedicated clinics; however, rates of this wound’s recurrence are high (Barwell et al., 2004).

### Diabetic foot ulcers

**Fig. 2.**
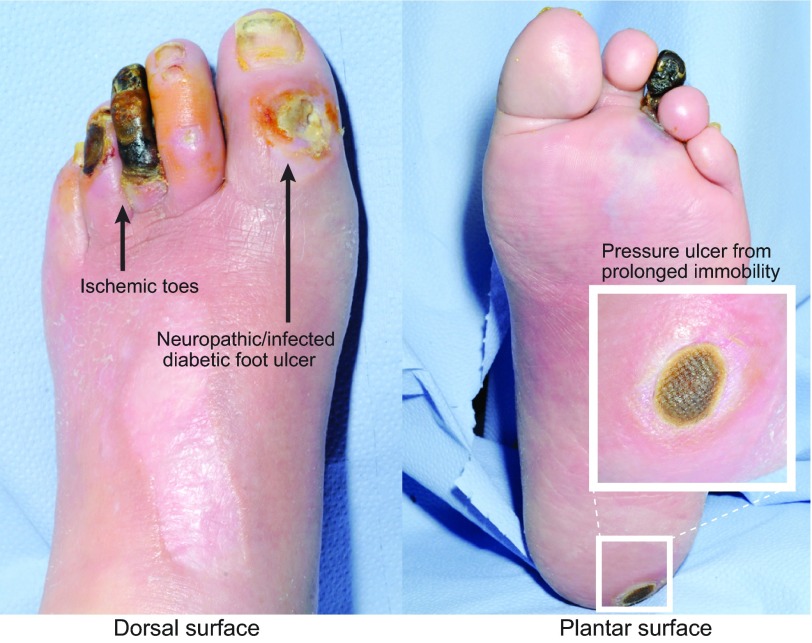
**Three classes of chronic wound on a diabetic patient’s foot.** This image shows a diabetic individual with three classes of chronic wound on the same foot: an ischemic third toe, a neuropathic/infected diabetic foot ulcer (DFU) on the big toe and a pressure ulcer (PU) on the heel from prolonged immobility.

A total of 15% of all individuals with diabetes will be affected by a foot ulcer (see case study in Box 2 and Fig. 2), generally in later life, and recurrence of these ulcers is seen in over 70% of patients within 5 years (Reiber et al., 1998; Apelqvist et al., 1993). Diabetes is a systemic disease that causes neuropathy and arterial damage, affecting many tissues and organs. Arguably, diabetic foot disease has the greatest clinical impact of all the medical complications associated with diabetes; indeed, 85% of diabetic limb amputations are preceded by an ulcer (Pecoraro et al., 1990). Neuropathic foot ulcers are treated by ‘offloading’ or pressure redistribution to relieve persistent pressure to sites of injury. In many patients, ischemia is present and can also be treated with drugs or surgery to improve arterial flow to the foot. Often, microbial infection of these ulcers is a problem and can spread to the bones of the foot. As such, the management of infection is a crucial component of DFU treatment (Jeffcoate and Harding, 2003).

**Box 2. Case study**

**Fig. 3.**
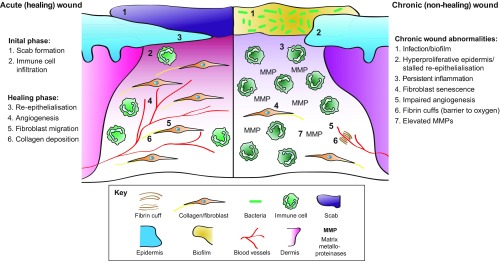
**The cellular and molecular differences between acute healing wounds and chronic non-healing wounds.** The healing of acute wounds (left) initiates with a transient inflammatory response as granulation tissue is formed, which provides an environment suitable for the re-epithelialisation required to complete repair. Chronic non-healing wounds (right) are often infected and exhibit persistent inflammation. By definition, re-epithelialisation has stalled but is hyper-proliferative. Granulation tissue is sub-optimal with elevated matrix metalloproteinases (MMPs) present together with poor fibroblast and blood vessel infiltration. Fibrin cuffs can also be present that prevent the diffusion of oxygen through the wound, rendering it hypoxic.

Mr Jones (64 years old) has had type 2 diabetes for 15 years, which is complicated by ischemic heart disease. Over the past 6 months, his renal function has started to deteriorate. He presents to the clinic with a rapid deterioration in the condition of his left foot, including changes in colour to the third and fourth toes over the past month. He also has an ulcer on the dorsum of his big toe, which has developed over the past few weeks from wearing a new pair of shoes (Fig. 3). Leg pain had reduced his mobility, resulting in his sleeping in a chair. As a consequence he has noticed the development of a lesion on the lateral aspect of his left heel.

The lesions on his third and fourth toe show tissue necrosis due to arterial disease in the peripheral vasculature. The lesion on the dorsum of his first toe is due to a combination of ischemia and neuropathy, and it would appear that this ulcer is infected. There is bone palpable in the base of this toe, so he is at risk of developing osteomyelitis, which could require a combination of both surgical and medical treatment. His third toe is irretrievable and this toe could be amputated surgically. The lesion on his heel is due to pressure damage as a result of his inability to walk because of ischemic disease, and if this ulcer were to heal it would take many months. This limb obviously has a substantial degree of arterial insufficiency, and a case could be made in the near future for this patient to undergo amputation, below or above the knee, depending on the location and severity of the arterial insufficiency. Infection is very common in patients with diabetes and in patients with ischemic disease. Poor blood supply restricting delivery of antibiotics will make treatment of this infection extremely challenging. Our current state of knowledge of diabetic foot disease, and of infection in diabetic foot disease, does not provide clear guidance on optimal antibiotic choice, duration of treatment or route of administration. The fact that this patient has cardiac and renal complications due to diabetes illustrates the multi-organ nature of diabetic complications, and as such this patient has a considerably reduced life expectancy.

### Pressure ulcers

PUs (Fig. 2) are commonly seen in hospitals and in residential care homes in individuals who are old and frail and/or immobile, or have spinal cord injury. They are caused by a combination of persistent direct pressure (or repetitive cycles of pressure, leading to reperfusion injury) along with shear forces (acting in parallel to the body’s surface) and an impaired skin condition (Grey et al., 2006b). In a study across 25 hospitals in five European countries, 18.1% of patients had a grade 1–4 PU [see ‘Wound grading system (EPUAP)’ in Box 1], primarily found on the sacrum and heels (Vanderwee et al., 2007). It is estimated that 4% of UK health expenditure is spent on managing PUs, and UK clinical practice focuses on strategies to prevent and treat PUs, including appropriate mattresses and the turning of immobile patients (Bennett et al., 2004). Where these approaches fail, more aggressive interventions are required, including debridement (via scraping with scalpel or larval therapy that removes devitalised tissue) and, if infected, antibiotics.

Although chronic wounds will generally have a single classifiable cause, no two wounds are the same and this, in part, has made clinical trials investigating the effectiveness of therapeutic interventions very difficult (Gottrup and Apelqvist, 2012). Moreover, resolving the underlying cause will not necessarily lead to healing; for example, correcting the venous abnormality that causes hypertension invariably fails, on its own, to result in improved VLU healing rates, although it does reduce recurrence rates (Barwell et al., 2004).

Several major variables compound the difficulties of planning the best treatment regime and of designing clinical trials for testing chronic wound therapeutics. For example, these lesions present to the clinic at varying times after their onset, in individuals with a wide age range and various co-morbidities, and with differing (and complex to analyse) microbial loads, which can impact considerably on healing efficacy. In addition, together with such wounds, there is considerable variability in the patient’s cooperation with any management regime (Harding et al., 2002). In a specialist wound-healing clinic, up to 70% of chronic wounds can be encouraged to heal within 3 months, but it is very difficult to judge at the outset which will be the ‘treatable’ wounds; for those stubborn non-healing wounds, there are currently no research-driven treatment options.

Good animal models of chronic wounds will provide new opportunities to identify prognostic gene signatures of ‘healer’ versus ‘non-healer’ chronic wounds, and to identify potential therapeutic target pathways for ‘kick starting’ delayed or stubborn healing. In this article, we discuss the major perceived differences between acute and chronic wounds, and how the current animal models of chronic wounds might be optimised to reveal potential therapeutic targets for improving healing.

## Chronic and acute wounds: a comparison

A standard acute wound in a healthy individual follows a fairly reproducible time course with several overlapping phases that involve the mobilisation of different, key cell lineages (Fig. 3). After the initial formation of a wound clot, wound re-epithelialisation commences from about 12- to 18-hours post-wounding. This process begins at the wound’s edges and from stumps of cut appendages – most often hair follicles – providing the wound is not so deep as to have completely lost these structures (Martin, 1997; Gurtner et al., 2008). In parallel with epidermal movement over the denuded wound, and initiating with a similar time course, a granulation tissue is also established. This occurs in part through invasion of the wound by dermal fibroblasts at the wound margin, some of which convert to more contractile myofibroblasts, with bone-marrow-derived mesenchymal stem cells also contributing to the granulation tissue (Sasaki et al., 2008). This tissue is then invaded by a dense network of capillaries, which leads to its granular appearance, and it contracts to leave a smaller surface area for re-epithelialisation. This contraction and the subsequent aberrant deposition of collagen lead to scar formation. Throughout the repair process, an orchestrated influx of inflammatory cells is drawn to the wound: first neutrophils, recruited within the first hours after wounding, to kill invading microbes; and, subsequently, macrophages to guide the repair process and clear away, by phagocytosis, any spent neutrophils and other cell and matrix debris from the site (Eming et al., 2007; Eming et al., 2009). In a non-infected wound, these inflammatory cells die on site or leave the wound. With their resolution, and the retraction or death of wound vessels and myofibroblasts, a relatively normal skin patch is reestablished. A small cut can be closed and the scab lost within a few days to a week, and will leave behind a minimal scar and potentially a minor pigmentation mark, indicating where the wound had been (Levesque et al., 2013; Chou et al., 2013; Chadwick et al., 2012).

### Physical and molecular hallmarks of a chronic wound

Chronic wounds, by definition, do not follow this sequence of events (Fig. 3). As an example, histological studies of chronic VLUs show a characteristic piled up and hyperproliferative epidermal edge, abutting an ulcer base that is covered with exudate loaded with necrotic debris. Where there should be wound granulation tissue, there are vessels surrounded by fibrin cuffs (presumed to be a response to venous hypertension) and very little vessel sprouting, such that most chronic wounds are considered to be poorly vascularised; there are few, if any, myofibroblasts, but a heavy inflammatory infiltrate, particularly of neutrophils (Herrick et al., 1992). Frequently, hyperpigmentation as a consequence of melanocyte recruitment can occur at the wound site, and can persist even after a chronic wound has successfully healed.

At a molecular level, it seems that keratinocytes at the edge of a chronic wound express a gene signature that reflects their partial proliferative activation, including the upregulation of several cell cycle genes, among them the cyclins, and the suppression of cell-cycle-checkpoint regulators and p53; this might explain the epidermal hyperproliferation that is seen at ulcer wound edges (Stojadinovic et al., 2008). The fibroblasts of an ulcerated wound are seemingly senescent, have diminished migratory capacity (Brem et al., 2007) and are somewhat unresponsive to the migratory stimulant transforming growth factor-β (TGF-β) (Pastar et al., 2010). This is reflected in dramatically reduced levels of TGFβR, and in reduced levels of the downstream components of the TGFβR signalling cascade, as seen in biopsies of non-healing ulcer tissue. TGF-β is also known to inhibit epidermal proliferation and, thus, the downregulation of TGFβ signalling might also contribute to epidermal hyperproliferation (Pastar et al., 2010; Glick et al., 1993). An additional explanation for reduced growth factor signalling and responsiveness might lie in the increased levels of tissue-degrading matrix metalloproteinases (MMPs) seen in chronic versus acute wound-tissue fluids (Trengove et al., 1999). Potentially more informative for prognostic purposes than comparisons of chronic versus acute wounds are comparisons of healing versus non-healing chronic wounds. One transcriptional profiling study, which demonstrates the potential of this comparative approach, found significant downregulation of the wound-associated keratin (keratin 16) and its heteropolymer partners (keratin 6a and 6b) in the non-healing wound group (Charles et al., 2008).

### Chronic inflammation

Chronic, persistent inflammation is a hallmark of most chronic wounds (Loots et al., 1998; Diegelmann, 2003), whereas, during acute healing, the inflammatory response is normally resolved. Of course, it is difficult to distinguish whether a long-term open wound and its ongoing exposure to microbes causes the chronic inflammation, or *vice versa*, or both. In some chronic wound scenarios, the ongoing presence of certain immune-cell types can prove to be beneficial; for example, increased numbers of Langerhan cells in the epidermis of DFUs has been associated with better healing outcomes (Stojadinovic et al., 2013). However, in general, a large influx of innate immune cells into chronic wounds, and their retention there, is likely to inhibit many repair processes (Mirza et al., 2014). Even some of the useful functions of immune cells might be disrupted in chronic wounds: it seems that their bactericidal and phagocytic activities are reduced, in comparison to those in an acute wound setting (Naghibi et al., 1987; Park et al., 2009; Khanna et al., 2010). One consistent obstacle in the healing of many chronic wounds is a build-up of necrotic debris at the wound edge, which perhaps occurs as result of the reduced phagocytic capacity of immune cells at a chronic wound. As a consequence, it is often clinical practice to debride the wound, either mechanically or using maggots (fly larvae), to establish a ‘fresh new’ wound, which can lead to efficient re-epithelialisation (Gottrup and Jørgensen, 2011). Another indication that the epidermis that surrounds a chronic wound is not inherently incapable of healing comes from the observation that wounds caused by biopsies at chronic wound margins heal as efficiently as a standard acute wound (Panuncialman et al., 2010).

### Wound microflora

Early studies of the microflora associated with chronic wounds depended on culturing material obtained from wound swabs, but this approach excluded the vast numbers of microbes that do not thrive in culture. With the growth of microbiome 16S rRNA sequencing opportunities, it is now possible to survey the full microbial flora of wounds. Early datasets have revealed that diabetic and venous leg ulcers share some microbial genera in common, but some significant differences have been reported as well, with the microbial community from different PUs being the most variable (Smith and Kavar, 2010; reviewed in Grice and Segre, 2012). Almost certainly, some of these pathogens, and even excessive numbers of some otherwise commensal species, might hold the key to modulating the efficiency of healing, either directly by their actions on keratinocytes or wound fibroblasts, or indirectly by modulating the inflammatory response. A near-future goal should be to conduct a similar characterisation of fungal and viral infections in chronic wounds.

## Current models of chronic wounds

**Fig. 4.**
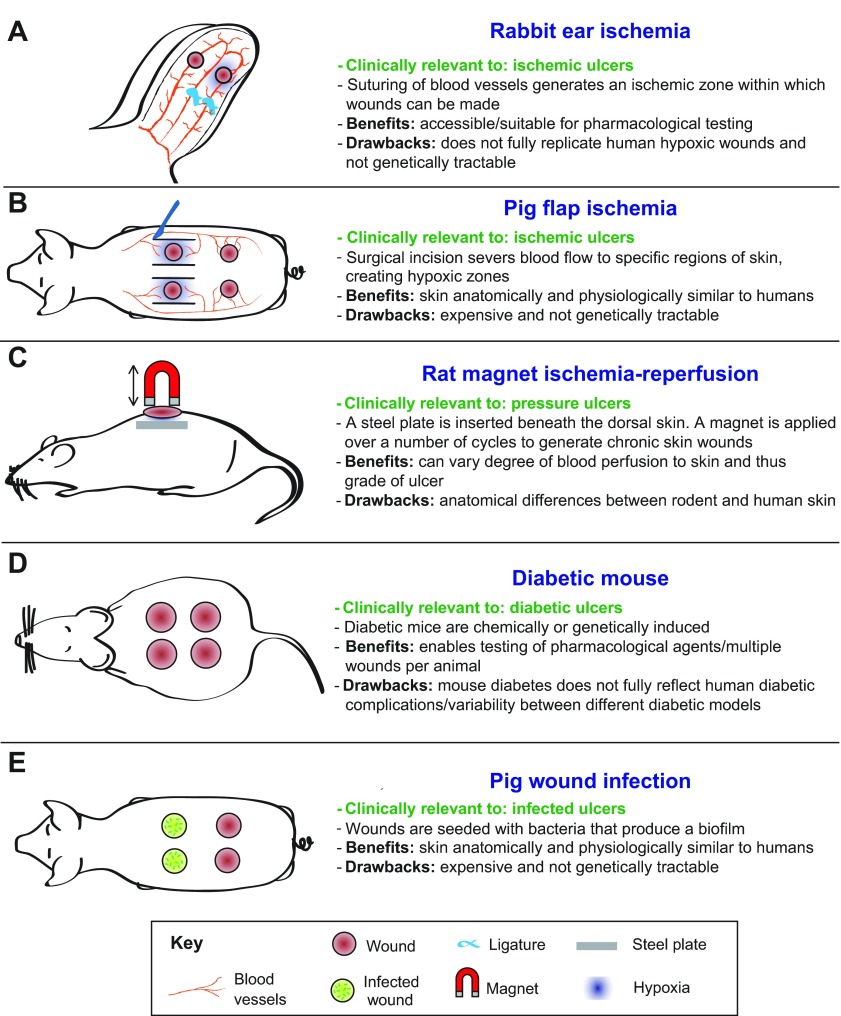
**Chronic-skin-wound animal models.** (A–E) Examples of chronic-skin-wound animal models, their clinical relevance, benefits and drawbacks. (A) Rabbit ear ischemia model (profile view). (B) Pig flap ischemia model (dorsal view). This method is also applicable to rodents and rabbits. (C) Rat magnet ischemia-reperfusion model (profile view). This method is also applicable to mice. (D) Genetically induced type 2 diabetic mouse model (dorsal view). (E) Pig wound infection model (dorsal view). This method is also applicable to rodents and rabbits.

Most animal models of chronic wounding are created by subjecting an acute wound to the primary clinical causes of chronic wounds, including ischemia, diabetes, pressure and reperfusion damage (Fig. 4). Below, we discuss the range of these chronic wound models and how they have furthered our understanding of chronic wound repair. But, because none of these models is optimal, we also discuss their limitations and how they could be improved to better aid our search for prognostic markers and therapeutic targets to promote healing.

### The rabbit ear ulcer model

The rabbit ear ulcer model (see Fig. 4A) is designed to generate an ischemic wound. An ischemic zone is created by suturing off two of the three arteries that supply the ear before making punch biopsy wounds down to the cartilage to create a full-thickness wound that lacks a vascular base and has very little lateral vascular supply (Ahn and Mustoe, 1990). This model has been used to test the potential therapeutic benefit of applying various growth factors to chronic wounds, including platelet-derived growth factor B (PDGF-B) (Liechty et al., 1999), keratinocyte growth factor 2 (KGF-2) (Xia et al., 1999) and vascular endothelial growth factor (VEGF) (Corral et al., 1999). One of these studies showed that the adenoviral delivery of the *PDGF-B* transgene to open ischemic wounds led to their healing even faster than non-sutured control wounds (Liechty et al., 1999), encouraging trials of this growth factor for enhancing chronic wound healing in the clinic. Although rabbits are inexpensive to maintain, breed prodigiously and are suited to testing potential therapeutics, they are limited in their genetic tractability, which hinders investigations into the underlying genetic basis of wound healing.

### Flap models to generate ischemic wounds

Skin flap models induce ischemia with or without necrosis, depending on the severity of perfusion occlusion or on the inclusion of silicone sheets to inhibit vascular in-growth from underlying vessels (Chen et al., 1999; Reid et al., 2004). Molecular markers have been used to validate the hypoxic skin wound model. Akin to human chronic wounds, rat hypoxic wounds, made on a bipedicle flap (surgically isolated area of skin with minimal continued blood supply) exhibit increased inflammatory markers and MMP activity (Chen et al., 1999). In pig (Fig. 4B), rat and mouse bipedicle skin flap models, wounds made in non-necrotic ischemic zones heal slower than those made on normally perfused skin (Chen et al., 1999; Roy et al., 2009; Biswas et al., 2010) and therefore can be used to establish mechanisms underlying ischemic wound healing. However, although porcine skin offers a closer anatomical comparison to human skin than loose-skinned rodents, and heals primarily through re-epithelialisation with minimal contraction, the cost involved in the breeding and maintenance of pigs poses a formidable barrier to research, as does their poor genetic tractability. This renders porcine studies practically more suited to preclinical studies investigating the effectiveness of therapeutic interventions, with rodent models geared towards understanding the fundamental genetic, cellular and molecular mechanisms of ischemic wound healing.

Severe ischemia can be induced by the transverse rectus abdominal myocutaneous (TRAM) flap method, which involves disrupting either the superior or inferior epigastric vessels. This operation generates necrotic skin wounds within 1 week (reviewed in Fang and Mustoe, 2008). However, this technique is limited by technical difficulty and the inability to perform more than one wound per animal. These studies are also most frequently performed in rats, rather than mice, owing to the larger size of rat blood vessels (therefore reducing the possibility for genetic manipulation because rats are not as genetically tractable as other model organisms). The TRAM method irreversibly obstructs blood supply to the skin; however, techniques that allow stop and start of blood flow would be more clinically relevant.

### Ischemia-reperfusion wounds

Animal models that allow wounded tissue to be reperfused with blood following hypoxia might better recapitulate human PUs or chronic wounds in which perfusion has been restored. The reperfusion of ischemic tissue is crucial for survival, but is known to cause secondary tissue damage through inflammatory mediators and the release of free oxygen radicals. Ischemia-reperfusion (IR) is currently modelled in two ways. Firstly, magnet IR (Fig. 4C) involves surgically implanting a metal plate under the skin of a loose skinned animal, such as a rat, followed by periodic compressions of the skin using an external magnet (Peirce et al., 2000; Wassermann et al., 2009). By varying the number and duration of these compressions, the size and extent of injury can be controlled to replicate certain features of human chronic wounds, including reduced blood flow, hypoxia and immune cell influx. Alternatively, in the mouse, a dorsal skin flap served by a single pedicle can be periodically clamped to generate an ischemic patch of skin that is also close to true clinical ischemia (Tatlidede et al., 2009). This latter method has yet to be combined with an acute skin wound, but both techniques show promise for investigations into the cellular and molecular processes that underlie the causal factors behind human PUs through their capacity to replicate the clinical cycles of compression-triggered hypoxia and reperfusion damage.

### Wounding diabetic mice

There are many animal models of diabetes (Fig. 4D) (Michaels et al., 2007; Chatzigeorgiou et al., 2009). Three of the most commonly used strains of mice to model chronic wound healing in individuals with diabetes are the Akita, NONcNZ010 and *db/db* mice. Alternatively, diabetes can be chemically induced in rodents, rabbits and pigs by streptozotocin (STZ), leading to death of pancreatic β cells (Michaels et al., 2007). In a direct comparison of these models, a recent study showed that the NONcNZ010 mouse model was most clearly impaired in its capacity to heal a splinted open wound (Fang et al., 2010), but all of these models can offer some insight into failed healing in diabetic tissues. One considerable problem in the field has been the variability in protocols used by laboratories (age of animal and type and/or size of wound), which makes comparing datasets very difficult.

In the last decade, a range of these models has been used to test whether the addition of exogenous growth factors might ‘kick start’ the healing process, and whether genetically deleting (‘knocking out’) various genes enhances or exacerbates impaired ‘diabetic’ wound repair. Moreover, researchers have begun to dissect the cell mechanisms that underpin failed healing and failed immune cell resolution, and these studies are beginning to offer real insight into potential therapeutics that might improve diabetic healing. Following activation, macrophages adopt a spectrum of activation states that extends from a pro-inflammatory/antimicrobial classical phenotype (M1) to a pro-repair/anti-inflammatory alternative phenotype (M2) (Gordon, 2003). However, this balance might be perturbed in diabetic individuals. *Lepr^db^* mice have been used to show that macrophages recruited to wounds in diabetic mice fail to polarise towards the M2 (alternatively activated), and this might lead to their retention at the wound site, and to enhanced MMP secretion and reduced collagen deposition (Bannon et al., 2013; Ploeger et al., 2013). Using the STZ-induced diabetic mouse model, it has now been shown that the recruitment of vascular progenitor cells to wound sites is retarded but can be rescued by a combinatorial therapy, involving hyperoxia (see Box 1) and SDF1α (stromal-cell-derived factor-1 alpha), which, respectively, promote increased circulating endothelial progenitor cell levels and their homing to wounds (Gallagher et al., 2007). Such studies offer real molecular insight and guide us towards potential therapeutic targets for eventual translation to the clinic.

### Limitations of current chronic wound models

Two aspects of the clinical chronic wound scenario that are poorly replicated in the models described above are ageing and the microbiota of the wound. There have now been some studies of ischemia-reperfusion wound models in aged mice (Park et al., 2009), and there is a considerable amount known about the role of systemic hormones and how they influence retarded healing in the elderly. It seems that falling oestrogen levels are the principal cause, in both males and females, of age-related healing impairment (Emmerson et al., 2012). Similarly, murine studies have shown that social isolation, leading to reduced cortisol levels and the associated lowering of KGF and VEGF at the wound site, leads to significantly retarded healing (Pyter et al., 2014). Because chronic wounds are most often suffered by the elderly, it would be beneficial to include this factor as a component of an optimal chronic wound model. Along these lines, several institutes are now developing ageing mouse colonies, which will enable the identification of ‘disease genes’ important in ageing populations. It would make good sense to layer ischemic and/or diabetic wound models onto such ageing screens to mimic the clinical scenario. Also important will be a degree of reverse translation; for example, as microbiota signatures emerge that are associated with the non-healing of human chronic wounds, this information should lead to the direct testing of how these particular combinations of microbes impact on host wound immune responses in either previously ‘sterile’ acute wounds or in chronic wound models in mice. Indeed, in infected wounds of diabetic mice, the changing profiles of microbiota coincided with delayed wound healing (Grice et al., 2010). However, caution is required when extrapolating murine inflammatory responses to humans. A recent study showed that, although global transcription responses and expression patterns in immune cells are largely conserved between the species, some specific genes show divergent expression (Shay et al., 2013). Unravelling the contribution of microbiota during wound healing is further complicated by the presence of bacteria-produced biofilms. Biofilm-producing bacteria known to colonise human chronic wounds inhibit re-epithelialisation in mouse wounds, which can be reversed through inhibiting biofilm production (Schierle et al., 2009). In porcine wounds infected with biofilms (Fig. 4E), wound closure rates were normal but they did exhibit marked differences in barrier function, suggesting that biofilms could lead to post-closure complications, including infections and the recurrence of skin breakdown (Roy et al., 2014).

Considering the heterogeneity and complexity of human chronic wounds, no single animal model is capable of fully recapitulating each clinical scenario. Care must be taken to choose the optimal animal model for each study, taking into account the benefits and limitations of each assay (including genetic tractability, reproducibility, cost and so forth). Several of the already existing models can be further developed by additional layering of other chronic wound causal factors, such as ageing, diabetes and infection status, to enable better modelling of the challenges facing the clinic.

## Unresolved questions and future challenges

Given our current knowledge of chronic wound pathology and the state of play with animal models, it is worth considering what the ultimate goals are for those of us working in this research area (see Box 3). In particular, what are the key questions that clinicians need answered in order to enable better therapeutic intervention, and which of these questions are realistically answerable with a targeted approach using currently available tools or minor modifications to those that we already have?

**Box 3. Clinical and basic research opportunities**

- When do chronic skin wounds stall?
- How does infection influence the healing process?
- Can the immune system be reprogrammed to restore healing?
- What are the commonalities across wounds that would enable one class of therapeutics to treat all wounds?
- Can angiogenesis be reactivated to restore normal oxygen tension to chronic wounds?

### When during repair does a chronic wound stall?

Almost all chronic wounds begin as a small cut or abrasion and almost certainly begin the repair process as if they were a normal acute wound. At some stage, the repair process then stalls but, of course, this is likely to be some days or weeks or even months before the patient presents at a clinic. Unfortunately, we have little understanding of the time or stage in the normal repair cycle when this stalling happens, and this could be crucial in developing therapeutics to reverse the failed process. There is a clear correlation between chronic wound duration and healing efficacy (Bosanquet and Harding, 2014), but more precise biomarkers to indicate key stages in the normal repair process would certainly be useful here.

### Does microbe load block healing and why does antibiotic treatment often fail?

If it turns out that the wrong cocktail of microbes in a susceptible wound is associated with, and even causes, failed healing, then we need to know whether this is entirely due to their effects on the host immune response and, if so, whether this cycle is reversible or not. In this regard, it is clear that antibiotics often fail to ‘cure’ an ulcer, although this might be because the systemic and/or topical delivery of antibiotics fails to reach the microbes owing to poor circulation and/or biofilm barriers.

### Can we reprogramme the immune response to kill pathogens and then resolve?

It is now well understood that innate immune cells exhibit various phenotypes or activation states, and can be either antimicrobial or enhance tissue repair by their release of growth factors and cytokines. Learning how to manipulate or to programme the inflammatory response so that it is most effective at staving off infection and then able to switch to repair mode, and finally then resolve in a timely fashion to avoid the chronic inflammatory phenotype common to chronic wounds, would be an important advance. One opportunity here might be to utilise the body’s own inflammation-resolution signals to drive early closure of the inflammatory response (Cash et al., 2014).

### What common features might aid in developing generic therapeutics?

Currently, the molecular mechanisms that underpin each type of chronic wound are not well understood and are seldom analysed together, despite the fact that most clinics see a full spectrum of chronic wound types. Clearly, commonalities, if they exist, should become our priority targets because these could be extrapolated most quickly to the clinic. These might include particular microbial load ‘signatures’ or host immune-response profiles that can be countered by a simple combinatorial antibiotic and anti-inflammatory treatment.

### How to enhance angiogenesis in a chronic wound?

One potential commonality across all chronic wound types is poor angiogenesis and the failure to form a normal wound granulation tissue. If this is causal of wound failure then therapeutics to enhance angiogenesis might produce good results, but might require more than the simple addition of vascular growth factors. Alternative bioengineering and stem cell strategies could include enhancing the hypoxia-sensing, and responsiveness, of local vessels and approaches for nudging recruited stem cells to switch to endothelial precursors at the wound site. Other interventions aiming to boost wound healing, such as living skin equivalents, synthetic scaffolds and growth-factor-releasing stem cells are entering or are already in Phase 3 clinical trials (Rennert et al., 2013).

## Conclusions

With more consideration of the clinical causes of chronic wounds, it should be possible to optimise animal models so that they better recapitulate the clinical hallmarks of this condition and allow us to more precisely dissect its pathological mechanisms. Such advances would help us to identify therapeutic targets and to develop screens for biomarkers that will allow us to better stratify patients for the most appropriate treatments, and to develop new medicines that help ‘kick start’ healing in these debilitating conditions.
